# Correlation between a low serum free triiodothyronine level and mortality of severe pulmonary tuberculosis patients

**DOI:** 10.1186/s12879-024-09099-1

**Published:** 2024-02-14

**Authors:** Yan Yang, Xiaoqing Huang

**Affiliations:** 1 Department of Respiratory and Critical Care Medicine, Hangzhou Red Cross Hospital, Hangzhou City, Zhejiang Province China; 2 Department of Tuberculosis Intensive Care Unit, Hangzhou Red Cross Hospital, Hangzhou City, Zhejiang Province China

**Keywords:** Severe pulmonary tuberculosis, Low T3 syndrome, Mortality

## Abstract

**Background:**

This study aimed to assess the relationship between a low serum free triiodothyronine (FT3) level and the mortality of severe pulmonary tuberculosis (TB) patients.

**Methods:**

We performed a retrospective study and reviewed the medical records of patients with severe pulmonary TB between January 2016 and June 2022. The patient demographics, Acute Physiology and Chronic Health Evaluation (APACHE) II score, survival or death at 28 and 90 days after hospital admission, and serum FT3 level were recorded. Bivariate regression analysis was performed to study the relationship between mortality and the FT3 level. The Kaplan–Meier method and the log-rank test were used to compare the survival rates between patients with different serum FT3 levels.

**Results:**

Our study included 495 severe pulmonary TB patients, with 383 (77.4%) patients having a low serum FT3 level. The low-serum FT3 group had high 28-day and 90-day mortalities. The patients who had died by 28 or 90 days after hospital admission had a low FT3 level. Survival analysis showed that the patients with a low serum FT3 level had a low probability of survival at 28 days and at 90 days after hospital admission.

**Conclusion:**

The serum FT3 level was correlated with the 28-day and 90-day mortalities in patients with severe pulmonary TB. The serum FT3 level should be monitored in these patients to help manage their disease.

## Background

Tuberculosis (TB) is a major public health problem and the leading infectious cause of death [1]. In 2022, the global TB death toll was 1.3 million. In China, the number of new TB cases was about 748,000, with an estimated TB incidence of 52/100,000. Meanwhile, the number of TB deaths was estimated as 30,000, and TB mortality was 2/100,000 in China [2]. Pulmonary TB is the most common type of TB. Affected patients often have various severities [3]. When complicated by hemoptysis, spontaneous pneumothorax, respiratory failure, or multiple organ dysfunction syndrome, the rapid progression of TB can lead to severe TB [4], which carries a high mortality and requires admission to an intensive care unit (ICU) [5]. Therefore, understanding the relationships between TB and its comorbidities could facilitate the clinical management of TB patients.

One of the comorbidities in patients with TB is dysregulated thyroid hormone levels [6]. TB has been proposed to be a risk factor for hypothyroidism, whereas hypothyroidism also increases the risk for TB infection [7]. Some medications, such ethionamide, prothionamide, and para-aminosalicylate sodium, can induce hypothyroidism during TB treatment [8]. Moreover, thyroid hormones can modulate the immune responses in the body. In addition, hypothyroidism can increase the risk of TB^6^. As early as the 1960s, abnormal thyroid hormone level changes, with the T3 reduction as the main abnormality, were reported in patients without a definitive thyroid disease, including patients in hunger or severe malnutrition. By the later 1980’s, a new concept, euthyroid sick syndrome, also known as the low-triiodothyronine (T3) syndrome, was proposed. Patients with low-T3 syndrome commonly have decreased serum free triiodothyronine (FT3), with no change or decrease in free thyroxine (T4) level and no significant change in TSH level. Among all types of decreased thyroid gland functions, low-T3 syndrome has a high prevalence in patients with serious illnesses [9–11]. Increasing evidence suggests that low-T3 syndrome is a strong predictor for adverse outcomes in patients with various illnesses. For example, Pan et al. have reported that low-T3 syndrome is associated with a short survival in patients with multiple myeloma [12]. Similarly, in patients with community-acquired pneumonia, low-T3 syndrome is strongly associated with ICU admission and the 30-day mortality [13]. Additionally, low-T3 syndrome also has been reported to be a prognostic indicator in patients after major surgery, trauma, or burn injury [14]. By definition, low-T3 syndrome refers to a low level of serum T3, with a normal level of thyroid-stimulating hormone (TSH) [10]. In patients with severe pulmonary TB, the clinical significance of a low serum T3 level but a normal TSH level is unknown.

Here, we report our retrospective cohort study investigating the relationship between a low serum free T3 (FT3) level and mortality in patients with severe pulmonary TB. The aim of this study was to provide a reference tool for clinicians so that they can pay attention to special TB patient groups in order to improve their treatment outcomes.

## Methods

### Study design and participant selection

We performed a retrospective cohort study and reviewed the medical records of patients admitted into the Department of Tuberculosis ICU at Hangzhou Red Cross Hospital, Zhejiang, China, between January 2016 and June 2022. The study protocol was approved by the hospital ethics committee.

The participant inclusion criteria were as follows: 1) ≥16 years old; 2) diagnosed with severe pulmonary TB based on the clinical presentation, laboratory tests, imaging examination, and pathological evaluations, according to the guidelines published by the National Health and Family Planning Commission of China [15]; 3) with an Acute Physiology and Chronic Health Evaluation (APACHE) II score of ≥8; 4) with no previous history of pituitary, hypothalamic, or thyroid diseases (hypothyroidism, hyperthyroidism, or thyroiditis); 5) with a normal serum TSH level; 6) not taking medications that might affect thyroid hormone production, such as levothyroxine, amiodarone, lithium, iodine-containing medications, glucocorticoids, ethionamide, prothionamide, and para-aminosalicylate sodium. Pregnant or breast-feeding patients as well as patients with incomplete medical records were excluded from the present study.

### Data collection

The medical records of the participants were reviewed. The demographics, APACHE II score, serum FT3 levels, and survival at 28 and 90 days after hospital admission were recorded.

All thyroid function tests were performed on the fasting peripheral blood samples obtained on the second day after hospital admission. The serum FT3 and TSH levels were tested by a thyroid hormone test kit in an automated chemiluminescence immunoassay analyzer (ADVIA Centaur XP, SIEMENS, Germany), according to the manufacturer’s instructions. The normal ranges for the serum FT3 and TSH levels were 3.5–10.4 pmol/L and 0.35–5.5 mIU/L, respectively.

### Statistical analysis

The patients were assigned into different groups based on their serum FT3 level. The continuous data are presented as the mean ± standard deviation or median with the interquartile range and compared by the Student’s *t* test or the Mann–Whitney test, depending on the normality test results. The categorical data are presented as numbers with percentages and compared by the Chi-squared test. The bivariate association and multivariate regression analyses were used to examine the relationships of the different serum FT3 levels with the patient survivals.

## Results

### Patients

A total of 495 patients (400 males and 95 females, aged 63.0 ± 20.3 years old) with severe pulmonary TB were included in the present study. There were 383 patients with a low serum FT3 level.

### Baseline characteristics of severe pulmonary TB patients with a low FT3 level

Most of the enrolled patients had a low FT3 level (77.4%). Compared with the patients with a normal FT3 level, the patients with a low FT3 level had a high APACHE II score (Table 1). Most of the patients with an abnormally low level of FT3 were in the age group of 60–89 years old (Table 2).

**Table 1:** Baseline characteristics of severe pulmonary tuberculosis patients with a low serum FT3 level

	FT3 level		
	Normal (*N* = 112, 22.6%)	Low (*N* = 383, 77.4%)	*P*
Sex, N (%)			0.683
Male	92 (82.1%)	308 (80.4%)	
Female	20 (17.9%)	75 (19.6%)	
Age, years, M ± SD	61.1 ± 21.0	64.0 ± 20.1	0.186
APACHE II, M ± SD	16.0 ± 7.6	22.2 ± 7.9	< 0.001*

*FT3* free triiodothyronine: *APACHE II* Acute Physiology and Chronic Health Evaluation II: *TSH* thyroid-stimulating hormone: *M ± SD* mean ± standard deviation

*, *P* < 0.05

**Table 2:** Distribution of severe pulmonary tuberculosis patients with a low serum FT3 in different age groups

Age group, years	FT3 (*N* = 383)
16–19	8 (2.1%)
20–29	30 (7.8%)
30–39	23 (6.0%)
40–49	21 (5.5%)
50–59	41 (10.7%)
60–69	74 (19.3%)
70–79	92 (24.0%)
80–89	80 (20.9%)
≥90	14 (3.7%)

### Mortalities among severe pulmonary TB patients with a low FT3 level

The 28-day and 90-day mortalities were significantly greater (*P* < 0.001) in the patients with a low FT3 level compared with those with a normal FT3 level (Table 3). Meanwhile, the patients who survived had a higher serum FT3 level than those who died within 28 and 90 days (Table 3). The degree of FT3 reduction was positively associated with the APACHE II score and disease severity.

**Table 3:** Association between the serum FT3 level and mortality in patients with severe pulmonary TB

	28-day mortality			90-day mortality		
	Survival	Death	*P*	Survival	Death	*P*
FT3	348	147	< 0.001*	(*N* = 308)	(*N* = 187)	< 0.001*
Low-FT3 group	247 (71%)	136 (92.5%)		212 (68.8%)	171 (91.4%)	
Normal group	101 (29%)	11 (7.5%)		96 (31.2%)	16 (8.6%)	
FT3 level (pmol/L)	3.08 ± 0.96	2.49 ± 0.76	< 0.007*	3.12 ± 0.76	2.56 ± 0.75	< 0.023*

*, *P* < 0.05

### Kaplan–Meier survival analysis

**Fig. 1:**
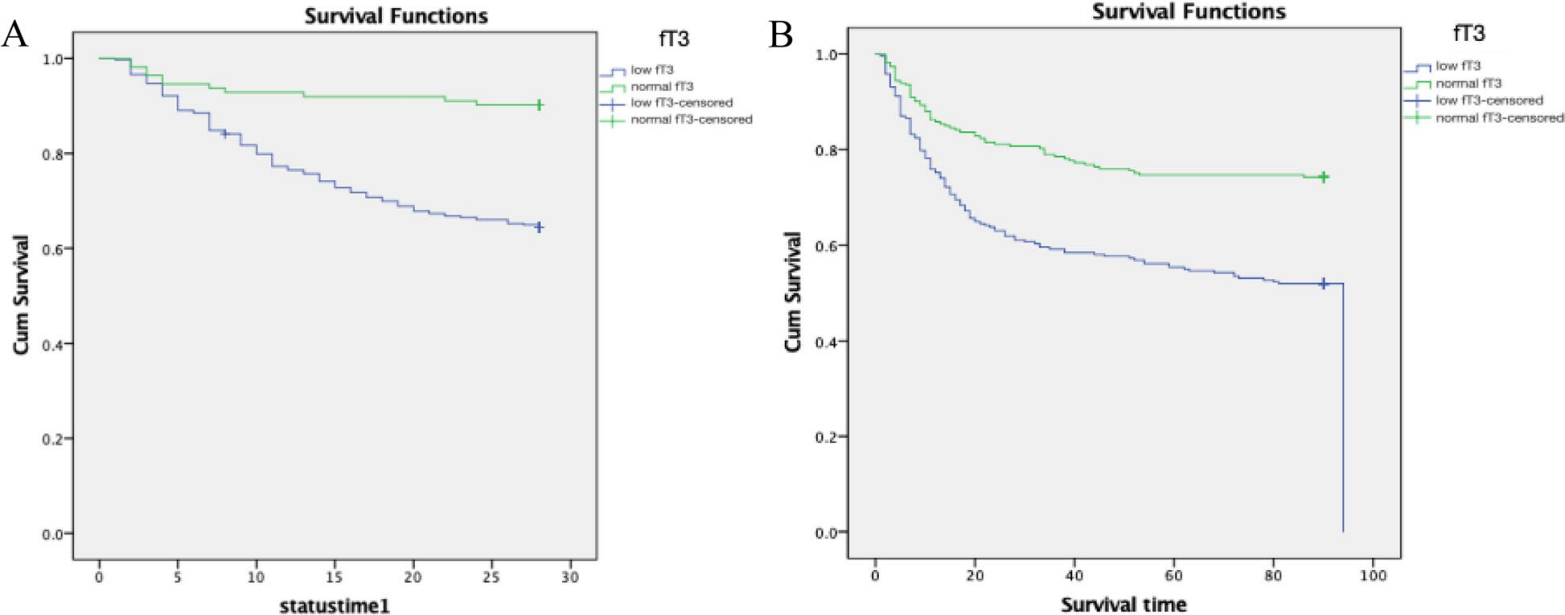
Kaplan–Meier survival comparisons. **A** Low vs. normal free triiodothyronine (FT3) at 28 days after hospital admission; **B** low vs. normal FT3 at 90 days after hospital admission. Green line, normal FT3. Blue line, low FT3

At 28 days and 90 days after hospitalization, the survival rate of patients with a normal serum FT3 level was greater than that of the patients with a low serum FT3 level. The differences were statistically significant (*P* < 0.001) between the patients with a normal vs. a low level of FT3 (Fig. 1).

## Discussion

There are limited previous studies on the clinical significance of a low serum T3 level in patients with severe pulmonary TB. In the present study, we studied the relationship between a low serum FT3 level with the mortality of patients with severe pulmonary TB. Our study results showed that the severe pulmonary TB patients with a low serum T3 level had a high mortality. The serum FT3 level was statistically lower in the death group than in the survival group. The findings of this study highlight the importance of monitoring the thyroid functions in these patients with severe pulmonary TB.

Thyroid hormone plays an important role in regulating various bodily functions [16]. The predominant thyroid hormone is T4, which is relatively inactive. T3, although it is secreted in a very small amount, is the active form of thyroid hormone [17]. In addition to primary thyroid disease, other serious illnesses, such as trauma, sepsis, infection, or strong stress, can all disturb thyroid hormone secretion and cause diseases [18, 19]. The pathogenesis of low-T3 syndrome is still unclear, but it is believed that the activity of 5′-deiodinase is reduced during severe stress, which results in decreased T4 deiodination in the peripheral tissues, a low T4 conversion rate, and finally a low T3 level [20]. In addition, patients with a low albumin level and a poor nutritional status can have reduced levels of thyroxine-binding globulin and thyroid transport protein, which leads to a low T3 level [21]. In patients with severe long-term illness, thyrotropin-releasing hormone is reduced, leading to decreased TSH secretion and ultimately a decreased serum T3 level [17].

The mean age of our patient population was 63.0 ± 20.3 years old. This finding was consistent with those of previous studies demonstrating that a low serum T3 level is more likely to be observed in the elderly population [22, 23]. With the aging process, the thyroid gland undergoes functional changes. Thyroid disorders, especially subclinical thyroid dysfunctions, are commonly reported in the elderly population due to chronic illnesses, malnutrition, and decreased metabolism and hormone production. In addition, elderly TB patients commonly have multiple comorbidities and underlying illnesses, which cause them to more easily develop severe TB [3]. Therefore, the thyroid function should be more closely monitored in elderly patients with severe TB.

Our analysis showed that the patients with a low serum T3 level had a high APACHE II score. In the clinic, APACHE II is a commonly accepted tool to measure disease severity [24]. A higher APACHE II score is correlated with a higher mortality. In the present study, the significantly higher APACHE II score in the severe pulmonary TB patients with a low serum T3 level suggested that the low T3 level correlated with the patient mortality. This finding was confirmed by our bivariate analysis, which showed that the 28-day and 90-day mortality rates were almost doubled in patients with a low serum T3 level compared to those with a normal serum T3 level. We also compared the serum T3 level in the survival group vs. the death group. The results also showed that the patients who died had a statistically significantly lower serum T3 level than those who survived. Furthermore, the survival analysis showed low survival rates of patients with a low serum T3 level. Altogether, these findings suggest a strong association between a low serum T3 level and mortality in severe pulmonary TB patients. This result was consistent with a previous report showing that low-T3 syndrome is an important risk factor for adverse outcomes in critically ill patients, probably due to the presence of proinflammatory cytokines, such as interferon gamma, tumor necrosis factor alpha, and interleukin-1 [25].

The strengths of our study were its large sample size and the fact that it is the first study to investigate the role of a low serum FT3 level in patients with severe pulmonary TB. The limitations of our study included its retrospective study design, which has inherent biases. We also were not able to clarify the temporal sequence of a low serum thyroid hormone level and TB occurrence, which might affect the mortality.

## Conclusions

In conclusion, our results suggest that a low serum FT3 level was associated with increased 28-day and 90-day mortalities in patients with severe pulmonary TB. Low-T3 syndrome is related with the severity and prognosis of patients. A lower serum FT3 level was associated with more severe disease and a higher mortality. In addition, serum FT3 reduction was also positively correlated with a more severe APACHE II score. Therefore, thyroid function tests should be performed at hospital admission to identify these severe pulmonary TB patients with a poor prognosis. Future prospective studies are required to confirm our study results as well as to investigate whether thyroid hormone supplementation can improve the mortality rates of these patients.

## Data Availability

The datasets generated and analyzed during the present study are available from the corresponding author on reasonable request.

## Abbreviations

APACHE: Acute Physiology and Chronic Health Evaluation
ICU: intensive care unit
TB: tuberculosis
T3: triiodothyronine
FT3: free triiodothyronine
TSH: thyroid-stimulating hormone
